# Gender differences in depressive-like behavior in Parkinson’s disease

**DOI:** 10.3389/fphar.2026.1961464

**Published:** 2026-10-21

**Authors:** Mannino Deborah, Cucinotta Laura, Basilotta Rossella, Labellarte Davide, Esposito Emanuela, Campolo Michela, Paterniti Irene

**Affiliations:** 1 Research Operative Unit of Neuropharmacology and Translational Neurosciences, Oasi Research Institute, Troina, Italy; 2 Department of Chemical, Biological, Pharmaceutical and Environmental Sciences, University of Messina, Messina, Italy

**Keywords:** BDNF/PI3K/AKT pathway, depression-like behaviours, gender difference, hippocampal synaptic loss, Parkinson’s disease

## Abstract

**Introduction:**

A Parkinson’s disease (PD) is a progressive neurodegenerative disorder characterized by both motor and non-motor symptoms, including depression, which markedly impairs patients’ quality of life. Although sex differences have been described in PD progression, the mechanisms underlying gender-specific susceptibility to depressive symptoms remain poorly understood.

**Methods:**

This study investigated sex-dependent differences in depressive-like behaviors and their underlying molecular mechanisms in an in vivo model of 1-methyl-4-phenyl-1,2,3,6-tetrahydropyridine (MPTP)-induced dopaminergic degeneration, using adult male and female CD1 mice.

**Results:**

Our results demonstrated that MPTP administration induced nigrostriatal degeneration and α-synuclein accumulation in both sexes, with more pronounced alterations in males. Male mice also exhibited greater depressive- and anxiety-like behaviors, accompanied by a marked reduction in 5-HT2C receptor expression, increased hippocampal acetylcholine levels, impaired expression of the synaptic markers PSD-95 and synaptophysin, and a stronger downregulation of the BDNF/PI3K/AKT pathway compared with females.

**Discussion:**

These findings demonstrate that sex significantly influences vulnerability to PD-associated depressive symptoms, with males showing greater behavioral and molecular impairment. Overall, our results highlight the importance of considering sex as a biological variable in PD research and support the development of personalized therapeutic strategies targeting non-motor symptoms.

## 1 Introduction

Parkinson’s disease (PD) is the second most common neurodegenerative disease worldwide, affecting millions of people, particularly those between the ages of 60 and 80 (Ou et al., 2021). Although PD has traditionally been defined by its motor symptoms (bradykinesia, rigidity, resting tremor, and postural instability) due to the progressive loss of dopaminergic neurons in the pars compacta of the substantia nigra and the depletion of striatal dopamine terminals, it is now widely demonstrated that non-motor symptoms are a fundamental component of the disease (Ramesh and Arachchige, 2023). These symptoms, often emerging in the early stages of PD, significantly contribute to disability and a reduction in the quality of life (Pena-Zelayeta et al., 2025). Among these, mood disorders, particularly depression and anxiety, are highly prevalent, with estimates suggesting that up to 50% of PD patients experience depressive symptoms during the disease (Alis et al., 2025). These emotional disturbances not only worsen patients’ daily lives but are also associated with a more rapid progression of both motor and cognitive decline (Reijnders et al., 2008). The pathophysiology of depression in PD is multifactorial and complex, involving dopaminergic degeneration, as well as alterations in several other neurotransmitter systems, including serotonergic, noradrenergic, and cholinergic systems (Jellinger, 2022). Specifically, serotonin (5-HT) dysfunction is thought to play a key role in the emotional dysregulation observed in PD, with altered 5-HT receptor expression contributing to the onset of depressive symptoms (Jellinger, 2022; Sancho-Alonso et al., 2025). Similarly, the cholinergic system, which is critical for mood regulation and cognitive function, is often compromised in PD, further exacerbating non-motor symptoms (Muller and Bohnen, 2013). The acetylcholine (ACH) system, particularly in regions such as the hippocampus, has been implicated in the cognitive decline and affective disturbances observed in PD patients, suggesting a potential target for therapeutic intervention (Haam and Yakel, 2017; Aosaki et al., 2010). Furthermore, the complex pathophysiology of PD-related depression involves impaired neurotrophic signaling, particularly within the brain-derived neurotrophic factor/phosphoinositide 3-kinase/protein kinase B (BDNF/PI3K/AKT) pathway, which plays a crucial role in mood regulation and neuronal survival (Martinowich et al., 2007). BDNF, a key regulator of neuronal survival, plasticity, and function, is significantly reduced in the brains of PD patients, particularly in areas such as the striatum and the prefrontal cortex (Angoa-Perez et al., 2017). Alterations in BDNF signaling may lead to neuronal atrophy and exacerbate depression, further highlighting the need for strategies aimed at enhancing neurotrophic support in PD (Numakawa and Kajihara, 2023). Despite growing consciousness of the impact of these non-motor symptoms, depression in PD often remains underdiagnosed and undertreated, highlighting the need for a deeper understanding of its underlying mechanisms and risk factors (Usnich et al., 2023). A particularly interesting aspect of PD concerns gender differences in the disease’s progression and symptomatology. Growing epidemiological evidence reported a higher incidence of PD in men than in women, suggesting a greater vulnerability to neurodegeneration in men (Cucinotta et al., 2024). Moreover, men generally develop more severe motor symptoms, such as rigidity and postural instability, while women exhibit a tremor-predominant phenotype (Kang et al., 2022). However, the gender differences in psychiatric outcomes, particularly in depression associated with PD, are not yet well investigated. Although men are generally more susceptible to neurodegeneration, women may have a neuroprotective response linked to estrogen, which could influence both motor and psychiatric symptoms (DuMont et al., 2023). Several animal models have been developed to study the pathophysiology of Parkinson’s disease (PD), including neurotoxin-based and genetic models. The 6-hydroxydopamine (6-OHDA) model selectively damages catecholaminergic neurons (Santoro et al., 2025), while the MPTP model induces dopaminergic neurodegeneration through its neurotoxic metabolite MPP+, affecting the nigrostriatal system (Li et al., 2026; Ardizzone et al., 2025). Genetic models provide complementary insights into the molecular mechanisms underlying PD (Frattini et al., 2025). In this study, we chose the MPTP model because it is a widely established approach in the literature for inducing nigrostriatal dopaminergic dysfunction and for studying nonmotor alterations associated with PD. This model therefore provides a suitable framework for studying sex-dependent differences in depression- and anxiety-like behaviors and the related neurochemical and molecular mechanisms (Liu et al., 2021). To further investigate the mechanisms underlying non-motor manifestations of PD, we analysed distinct brain regions. We assessed the nigrostriatal system for dopaminergic alterations using TH, DAT, and α-synuclein, and examined the hippocampus for changes in serotonergic and cholinergic neurotransmission, synaptic plasticity, and BDNF/PI3K/AKT signaling potentially associated with depressive- and anxiety-like behaviours. Therefore, this study aims to investigate gender differences in depression-like behaviours in a mouse model of MPTP-induced nigrostriatal degeneration. By exploring the neurobiological mechanisms underlying gender-specific susceptibility to depressive symptoms in PD, this research could provide crucial insights that could lead to more personalized and effective therapeutic strategies for PD patients.

## 2 Materials and methods

### 2.1 Materials

All other chemicals were of the highest commercial grade available. All stock solutions were prepared in non-pyrogenic saline (0.9% NaCl; Baxter, Rome, Italy).

### 2.2 Animals

Adult male and female CD1 mice (weighing 25–30 g and aged 10–12 weeks; Envigo, Udine, Italy) were employed in all experiments. Animals were housed (five per cage) in a controlled environment at 21 °C ± 1 °C, under a 12-h light/dark cycle. They had unrestricted access to standard laboratory feed and tap water. All procedures involving animals were conducted in accordance with Italian legislation on the use of animals for scientific purposes (Ministerial Decree 116/1992) and the European Council Directive (EEC) (Official Journal of the European Union L 358/1, 18/12/1986).

### 2.3 MPTP lesion

Mice were administered four intraperitoneal injections of MPTP (20 mg/kg; Sigma–Aldrich, St. Louis, MO, USA) in saline, spaced 2 h apart on a single day, resulting in a total dose of 80 mg/kg per animal. Fourteen days after the initial MPTP injection, all mice were euthanized by cervical dislocation, and brains were collected for histological and molecular analyses. The total MPTP dose of 80 mg/kg was selected based on previously reported *in vivo* studies (Lanza et al., 2023).

### 2.4 Experimental groups

Animals were randomly divided into the following four groups:

1. Sham Female: received vehicle via intraperitoneal injection (N = 10);
2. MPTP Female: received MPTP intraperitoneally on day one following the standard MPTP protocol (N = 10);
3. Sham Male: received vehicle via intraperitoneal injection (N = 10);
4. MPTP Male: received MPTP intraperitoneally on day one following the standard MPTP protocol (N = 10).

The minimum number of mice for every technique was estimated with the statistical test “ANOVA: fixed effect, omnibus one-way” with the G-power software. This statistical test generated a sample size equal to n = 10 mice for each technique.

### 2.5 Behavioral assessments

To evaluate motor and non-motor symptoms associated with PD following MPTP-induced lesion, a series of behavioral tests was conducted. These paradigms allow assessment of depressive-like and anxiety-like behaviors, which are common non-motor features of PD (Wei et al., 2021).

#### 2.5.1 Forced swim test

The forced swim test (FST) was conducted 14 days after MPTP administration to evaluate behavioral despair, another symptom associated with PD-related depression. Mice were placed individually in transparent glass cylinders (25 cm height × 10 cm diameter) filled with fresh tap water (25 °C ± 1 °C, 15 cm depth). The time spent immobile—defined as floating without struggling—during the last 4 min of a 6-min session was recorded. Increased immobility time is indicative of higher depressive-like behavior. After testing, mice were gently dried and placed near a heater for 20 min.

#### 2.5.2 Light/dark test

The light/dark test is a commonly employed behavioral assay for assessing unconditioned anxiety-like responses in rodents. This test exploits the natural conflict between a rodent’s tendency to explore new environments and its instinctive avoidance of brightly illuminated open spaces. Animals were placed in a two-chamber apparatus, comprising a dark, enclosed compartment and a brightly lit open compartment connected by a small opening. For analysis, the time spent in the light compartment was recorded over a 5-min session. Reduced time in the illuminated chamber, longer latency to leave the light area, and fewer transitions were interpreted as indicators of increased anxiety-like behavior.

#### 2.5.3 Sucrose preference test

The sucrose preference test was performed to assess anhedonia, a depressive-like symptom often observed in Parkinson’s disease. Animals were first acclimated to two identical water bottles in their home cages and trained to consume a 2% sucrose solution for 3 days to reduce novelty effects and establish baseline preference. After 18 h of food and water deprivation—during which only drugs or vehicle were administered—each animal was presented with two bottles (2% sucrose solution and tap water) for 1 h. The amount of each solution consumed was measured by weighing the bottles before and after the test. The percentage of sucrose preference served as an index of anhedonia, with reduced preference reflecting depressive-like behavior (Filippone et al., 2023).

#### 2.5.4 Elevated plus-maze test

The elevated plus-maze (EPM) was used to assess anxiety-like behavior 14 days after MPTP-induced lesion. The apparatus consisted of two open arms (45 × 10 cm) without side walls and two closed arms (45 × 10 × 30 cm) with side and end walls, extending from a central platform (10 × 10 cm). The maze was elevated 60 cm above the floor in a dimly lit room (∼8 lux at the center). Each animal was placed in the center facing an open arm and allowed to explore for 10 min. The number of entries and time spent in open versus closed arms were recorded and analyzed from video recordings. The percentage of open-arm entries and time spent in open arms were used as indicators of anxiety-like behavior, while the total number of arm entries measured general locomotor activity.

### 2.6 Stereological quantitation of tyrosine hydroxylase-positive neurons

Unbiased counting of tyrosine hydroxylase (TH) + dopaminergic neurons within the substantia nigra par compacta (SNpc) was performed as previously described (Ardizzone et al., 2025). The sections were incubated with polyclonal primary antibody mouse anti-TH (1:400, Santa Cruz Biotechnology) O/N. Brain sections were counterstained with cresyl violet, a Nissl stain. To count the number of TH + cells, StereoInvestigator software was used (Microbrightfield, Williston, VT). Cells were counted with a 10 × and 20 × objective, respectively, using a Nikon Eclipse Ci-L microscope. The area of interest for counting TH-immunoreactive cells was performed within a 50 × 50 × 5 µm frame on the same side of the brain, with an upper and lower control zone of 1 μm; for Nissl cell counting, the same sections were examined.

### 2.7 Immunohistochemical staining of dopamine transporter and α-synuclein

Immunohistochemical analyses were performed in the SNpc following previously established protocols (Mannino et al., 2023). Brain sections (7 µm thick) were used for staining and incubated overnight with the following primary antibodies: anti-dopamine transporter (DAT, 1:100; sc-14002, Santa Cruz Biotechnology), and anti-α-synuclein (α-syn, affinity-purified rabbit polyclonal antibody targeting the C-terminal region of human α-synuclein, 1:100; sc-7011, Santa Cruz Biotechnology, Dallas, TX, USA). Following primary antibody incubation, sections were rinsed with phosphate-buffered saline (PBS) and incubated with appropriate secondary antibodies for 1 h. The signal of binding with the antibodies was amplified by using the peroxidase Avidin–Biotin complex (Vector Lab. Inc., Burlingame, CA). The reaction was revealed by a chromogenic substrate (brown DAB) and counterstained with Nuclear Fast Red. Immunoreactivity % was quantified using the ImageJ software that counted the positive pixels. Images were obtained by using a Nikon Eclipse Ci-L microscope. Photomicrographs were evaluated densitometrically using Optilab software (Graftek, Mirmande, France) on a MacBook Pro (Apple, Cupertino, CA, USA). Staining intensity was scored on a scale from 0 to 10 (0 = no immunoreactivity; 10 = intense dark brown staining) by three independent, blinded observers. All sections were analyzed in a blinded manner. Representative images were acquired at 20× (50 µm scale bar) and 40× (20 µm scale bar) magnification.

### 2.8 ELISA kit for acetylcholine

Acetylcholine (ACH) levels were quantified both in hippocampal tissue extracts and in serum using a commercial ELISA kit (Mouse ACH ELISA Kit, MBS733116; MyBioSource), following the manufacturer’s instructions.

### 2.9 Double immunofluorescence for PSD-95 and SYP

Double immunofluorescence staining was performed in hippocampal sections to evaluate synaptic markers. Colabeling was carried out for MAP2/PSD-95 and MAP2/SYP. Following deparaffinization, sections were incubated overnight with the following primary antibodies: anti-microtubule-associated protein 2 (MAP2) (1:100; ab5622, Sigma-Aldrich), anti-PSD-95 (1:100; sc-sc-32291, Santa Cruz Biotechnology), and anti-synaptophysin (SYP) (1:100; sc-17750, Santa Cruz Biotechnology, CA, USA). On the following day, sections were incubated for 3 h with the appropriate secondary antibodies: Alexa Fluor 488 goat anti-mouse (A11001; Molecular Probes, Eugene, OR, USA) or Alexa Fluor 594 anti-rabbit IgG (A11008; Molecular Probes, Eugene, OR, USA), depending on the primary antibody. Nuclear counterstaining was performed with 4′,6-diamidino-2-phenylindole (DAPI) (Hoechst, Frankfurt, Germany). All analyses were performed in a blinded manner. Images were acquired by randomly analyzing the entire region of the hippocampus, with a Nikon Eclipse Ci-L fluorescence microscope and are shown at ×40 magnification.

### 2.10 Western blot analysis

For Western blot analysis, the hippocampus was isolated from brain tissues, following protocols previously standardized in our laboratory (Filippone et al., 2022). Cytosolic and nuclear protein fractions were isolated and separated by SDS-PAGE (10%–12%), followed by transfer onto membranes. The membranes were incubated overnight at 4 °C with the following primary antibodies: anti-serotonin 5-HT2C receptor (SR2C) (1:100; sc-17797, Santa Cruz Biotechnology), anti-postsynaptic density 95 (PSD-95) (1:100; sc-32291, Santa Cruz Biotechnology), and anti-synaptophysin (SYP) (1:100; sc-17750, Santa Cruz Biotechnology, CA, USA), anti-brain-derived neurotrophic factor (BDNF) (1:500; sc-65514, Santa Cruz Biotechnology), anti-phospho- serine/threonine kinase 1/Protein kinase B (p-AKT) (1:500; sc-514032, Santa Cruz Biotechnology), anti- AKT (1:500; sc-5298, Santa Cruz Biotechnology), and anti- Phosphoinositide 3-kinase (PI3K) (1:500; sc-365404, Santa Cruz Biotechnology). On the following day, membranes were incubated for 1 h at room temperature with appropriate HRP-conjugated secondary antibodies (anti-mouse or anti-rabbit, 1:1000; Jackson ImmunoResearch, West Grove, PA, USA). To ensure equal loading, cytosolic fractions were probed with β-actin (1:500; sc-47778, Santa Cruz Biotechnology). Protein detection was achieved using enhanced chemiluminescence reagents (ECL, ThermoFisher, Waltham, MA, USA). Densitometric analysis of the protein bands was carried out with Bio-Rad ChemiDoc™ XRS + software (Version 3.0.1), and results were normalized to β-actin levels.

### 2.11 Statistical analysis

Experimental data are expressed as mean ± standard deviation (SD) of N observations, in which N represents the number of animals studied. One-way ANOVA analysis of variance followed by the Bonferroni *post hoc* test for multiple comparisons was used to analyze the results, and only a *p*-value less than 0.05 was considered significant. Data are representative of a minimum of three independent experiments.

## 3 Results

### 3.1 Gender-related differences in pathological hallmarks of PD

To validate the experimental model, we first examined the hallmarks of PD in SNpc. Nissl staining revealed a significant reduction in TH in MPTP-treated mice compared to the sham group, particularly in male mice (Figures 1A–D). Similarly, immunohistochemistry showed a significant reduction in DAT in MPTP-treated female and male mice compared to sham mice (Figures 2F–I). The data also showed a significant accumulation of α-synuclein in MPTP-treated female and male mice compared to the respectively sham mice (Figures 1K–N). Comparing the results by gender, the alterations were slightly more pronounced in males than in females, confirming a greater predisposition of males to PD than females (Cerri et al., 2019).

**FIGURE 1:**
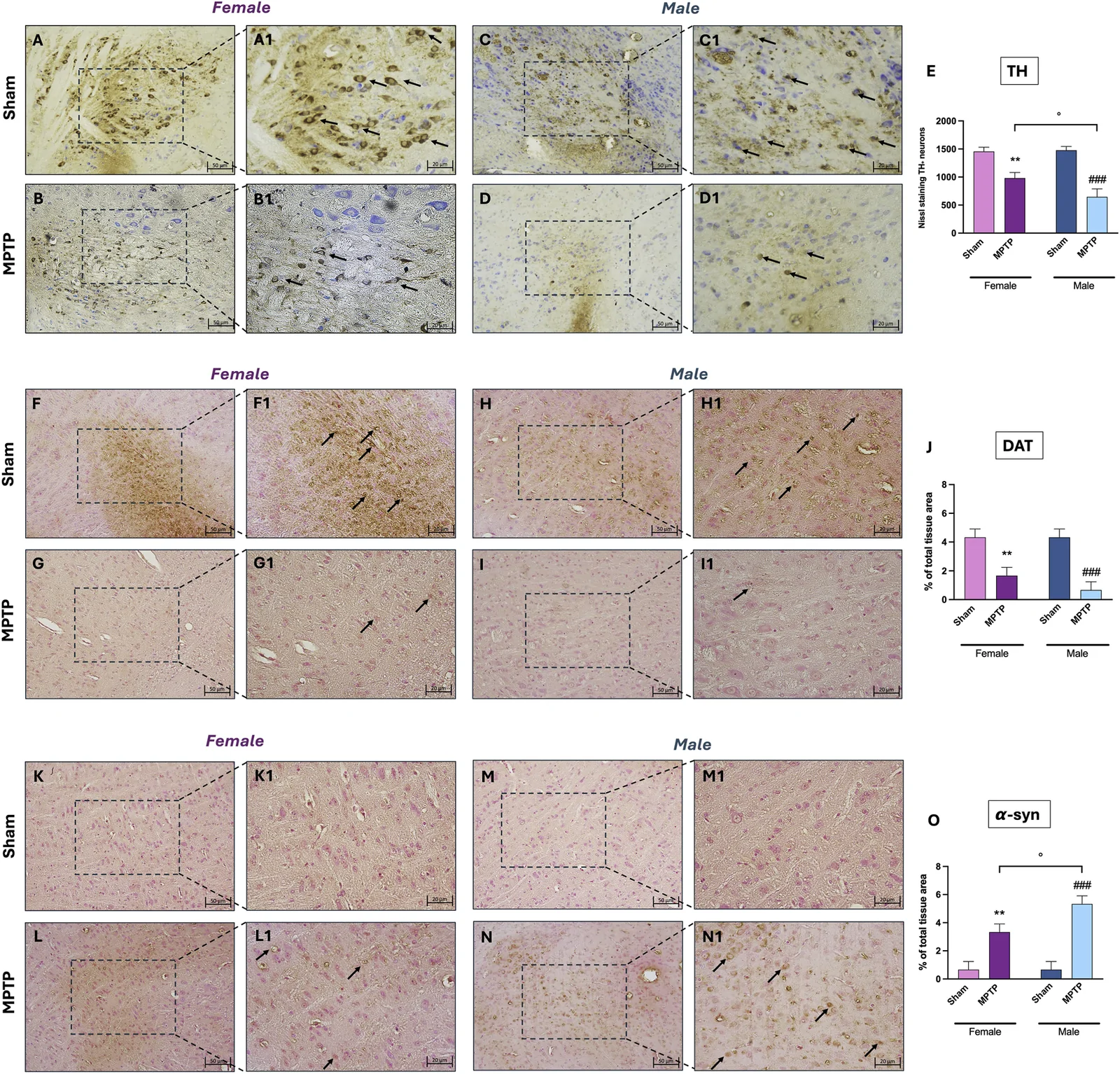
Evaluation of gender differences on PD’s hallmarks. Images of TH expression are shown: female [Sham **(A,A1)** and MPTP **(B,B1)**] and male [Sham **(C,C1)** and MPTP **(D,D1)**], score **(E)**. Images of DAT expression are shown: female [Sham **(F,F1)** and MPTP **(G,G1)**] and male [Sham **(H,H1)** and MPTP **(I,I1)**], score **(J)**. Images of α-synuclein expression are shown: female [Sham **(K,K1)** and MPTP **(L,L1)**] and male [Sham **(M,M1)** and MPTP **(N,N1)**], score **(O)**. Images are shown at ×20 and ×40 magnification. Data are expressed as mean ± SD. One-way ANOVA test. **p < 0.01 vs. Female Sham; ###p < 0.001 vs. Male Sham; ° p < 0.05 vs. MPTP.

**FIGURE 2:**
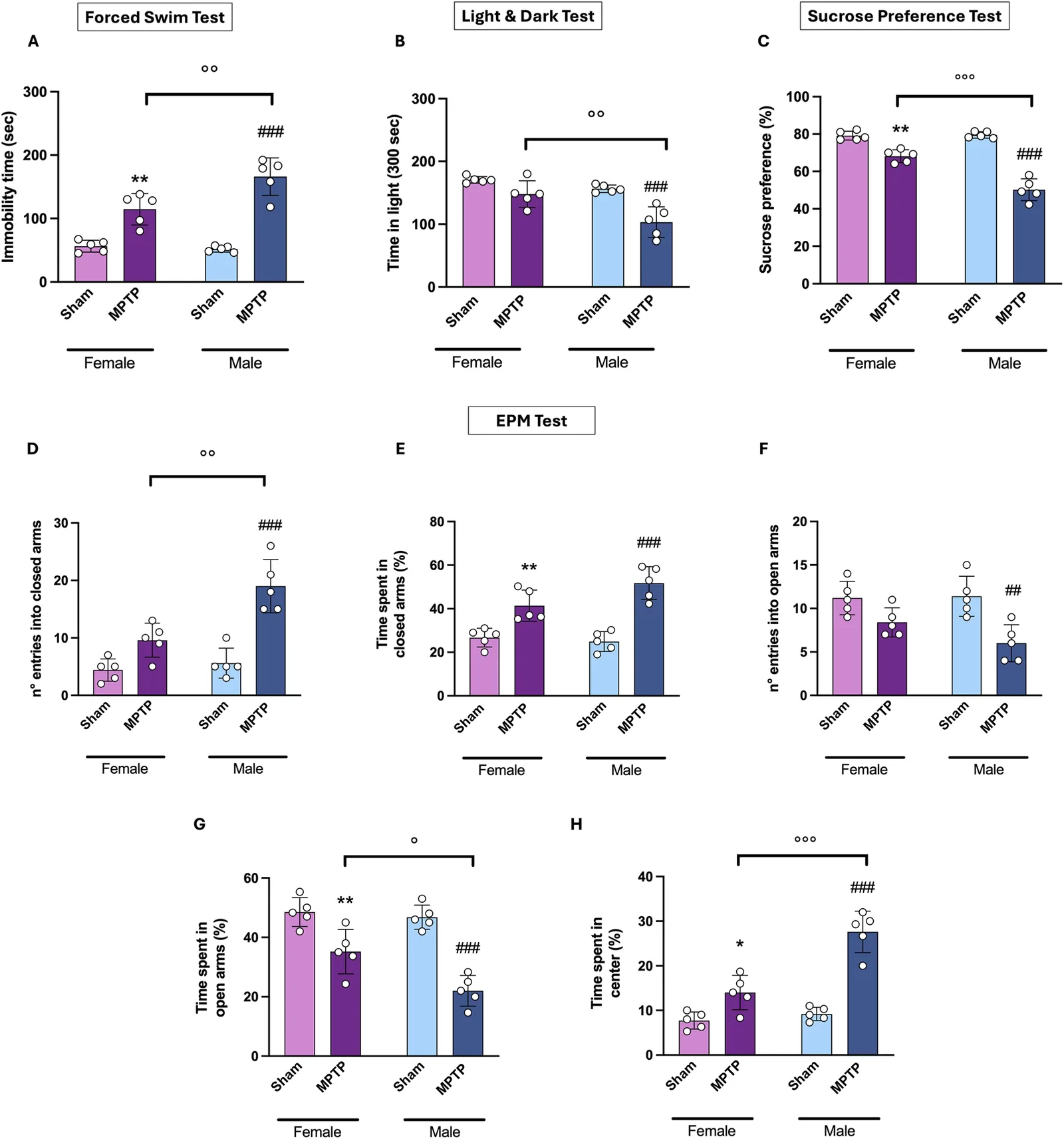
Gender difference on behavioural assessment. Forced Swim Test **(A)**, Light/Dark test **(B)**, Sucrose Preference Test **(C)** and EPM Test **(D–H)**. Data are expressed as mean ± SD. One-way ANOVA test. *p < 0.05 and **p < 0.01 vs. Female Sham; ##p < 0.01 and ###p < vs. Male Sham; °p < 0.05, °p < 0.01 and ° p < 0.001 vs. MPTP.

### 3.2 Gender differences in depressive-like behaviours in the MPTP model

Behavioural tests revealed clear gender-specific effects of MPTP. In the FST (Figure 2A), both male and female mice showed increased immobility compared to their respective sham groups, and MPTP-treated male mice exhibited significantly greater immobility time compared to females. Similarly, in the Light & Dark Test (Figure 2B), males spent less time in the light compartment than females. In the Sucrose Preference Test (Figure 2C), MPTP significantly decreased sucrose preference relative to sham in both sexes, and male mice showed a marked reduction in sucrose consumption, indicative of an anhedonic phenotype. Finally, anxiety-like behaviours were assessed using the EPM test (EPM, Figures 2D–H). Overall, MPTP exposure led to increased anxiety-like behaviours compared to sham animals, and MPTP-treated male mice spent more time in closed arms and in the centre, and less time in open arms, compared to female mice. These data suggested that male mice show increased susceptibility to MPTP-induced depressive and anxiety-like behaviours.

### 3.3 Gender-specific alterations in neurotransmitter systems in the MPTP-model

In PD, depression is associated with alterations in neurotransmitter systems, including serotonin, which plays a key role in mood regulation (Dalle and Mabandla, 2018), and acetylcholine, which has been implicated in depressive symptoms through its interaction with dopaminergic deficits (Lester et al., 2010). Western blot analysis revealed a significant reduction in SR2C expression in MPTP-treated animals compared to sham groups, particularly this reduction was pronounced in male mice (Figure 3A). Moreover, the ELISA assay revealed no change in serum acetylcholine levels, but a significant increase in hippocampal tissue acetylcholine levels following MPTP exposure compared to sham, especially in male mice (Figures 3B,C). These results suggest that gender-specific alterations in serotonergic and cholinergic signalling may contribute to the observed behavioural differences in MPTP model.

**FIGURE 3:**
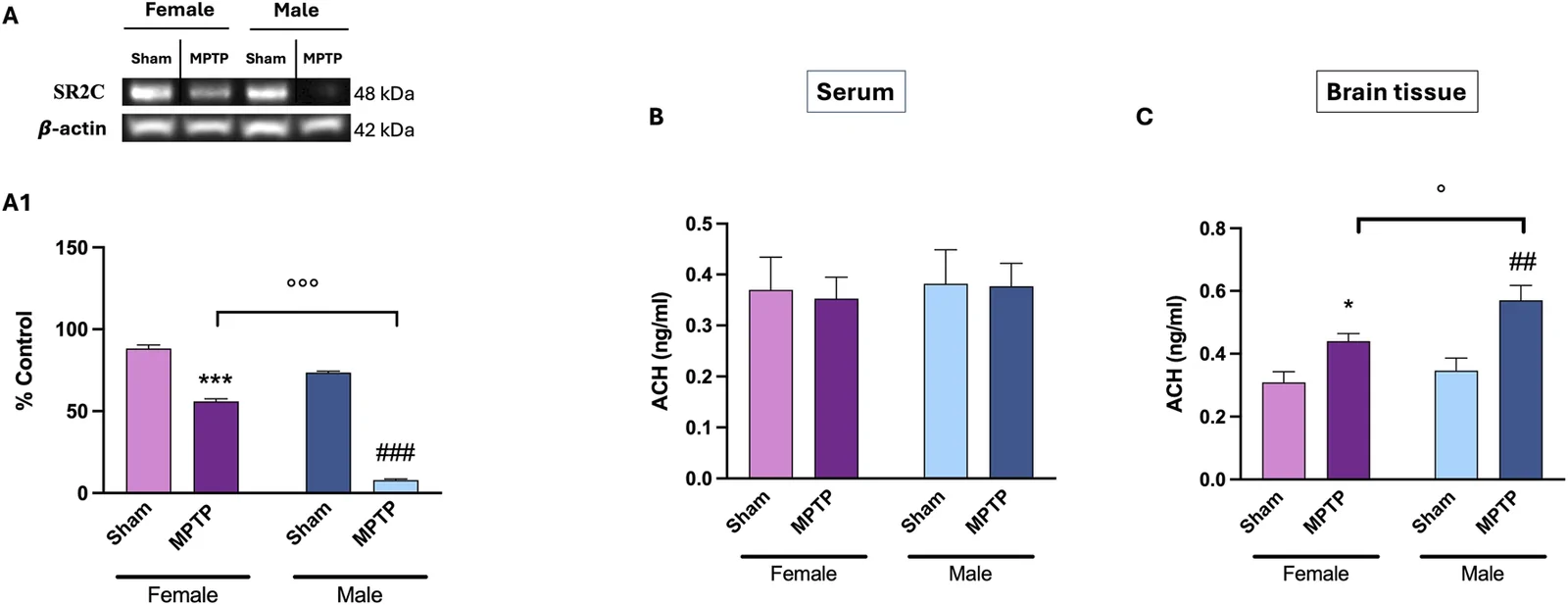
Gender difference on neurotransmitter alterations. Western blot data showed SR2C expression **(A, A1)** and ELISA assay for acetylcholine levels in serum **(B)** and in hippocampal tissue **(C)**. Data are expressed as mean ± SD. One-way ANOVA test. *p < 0.05 and ***p < 0.001 vs. Female Sham; ##p < 0.01 and ###p < vs. Male Sham; °p < 0.05 and °p < 0.001 vs. MPTP.

### 3.4 Gender-dependent impairment of hippocampal synaptic plasticity

Given the critical role of the hippocampal function in mood regulation (Tartt et al., 2022), we next examined synaptic integrity. Double immunofluorescence analysis was performed to evaluate the expression of PSD-95 and SYP co-labeled with MAP2. Data showed that MPTP exposure induced a significant reduction of both PSD-95 (Figures 4A–E) and SYP (Figures 4F–J) expression compared to sham groups, with these alterations being more pronounced in male mice, whereas female mice showed relatively preserved synaptic marker levels. These data indicate that MPTP compromises hippocampal synaptic plasticity in a gender-dependent manner.

**FIGURE 4:**
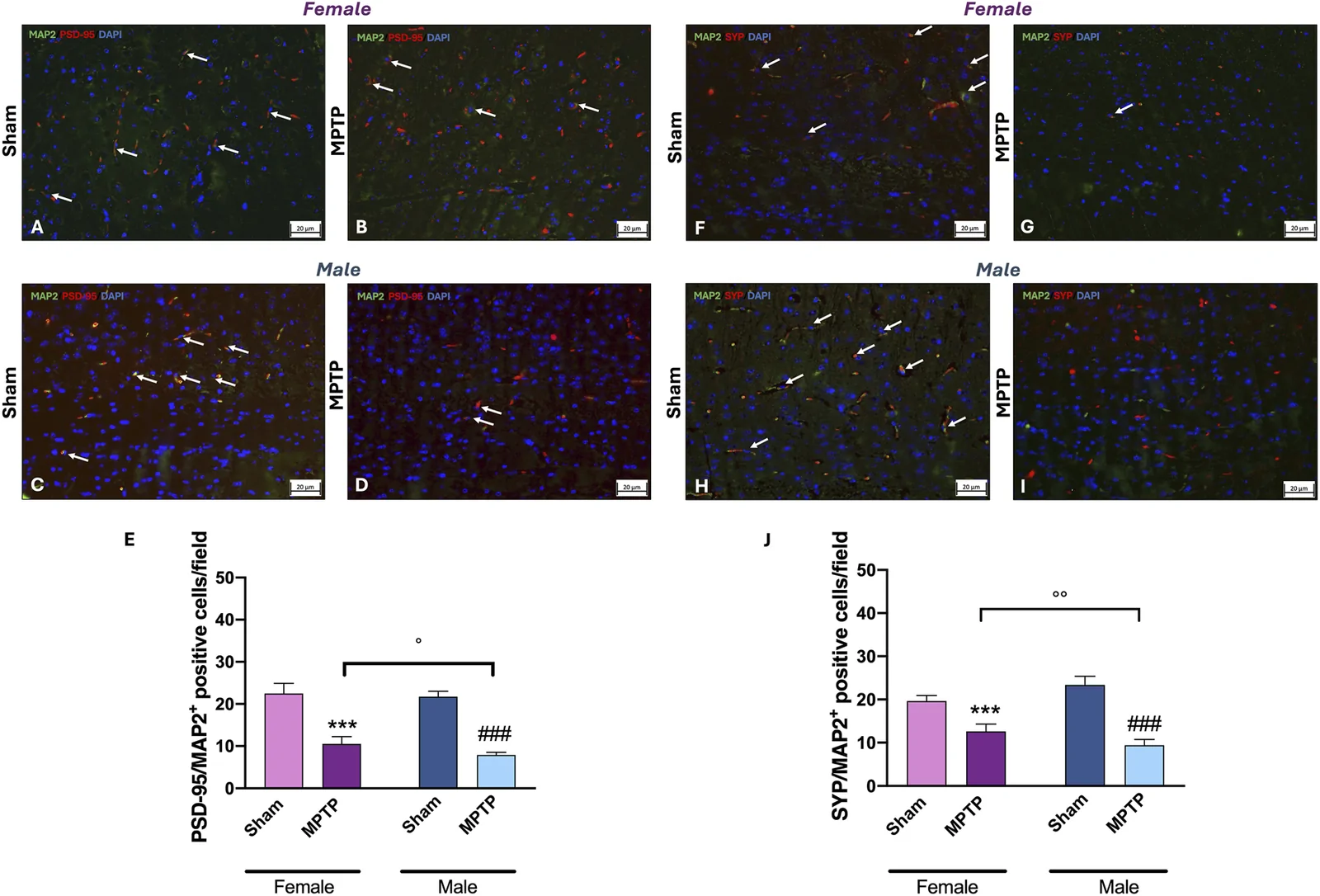
Gender-difference on hippocampal synaptic plasticity. Immunofluorescence co-label of PSD-95 and MAP2 in female [Sham **(A)** and MPTP **(B)**] and male [Sham **(C)** and MPTP **(D)**], score **(E)**. Immunofluorescence co-label of SYP and MAP2 in female [Sham **(F)** and MPTP **(G)**] and male [Sham **(H)** and MPTP **(I)**], score **(J)**. Images are shown at ×40 magnification. Data are expressed as mean ± SD. One-way ANOVA test. ***p < 0.001 vs. Female Sham; ###p < vs. Male Sham; °p < 0.05 and °p < 0.01 vs. MPTP.

### 3.5 Gender-specific modulation of the BDNF/PI3K/AKT pathway

Finally, we examined the BDNF/PI3K/AKT signalling pathway, which has been consistently associated with depressive disorders (Sun et al., 2022). Western blot analysis showed a marked reduction in BDNF levels (Figure 5A) as well as decreased activation of PI3K (Figure 5B) and p-AKT (Figure 5C) in MPTP-treated mice compared to sham groups in both sexes. These changes were more pronounced in male mice, whereas female mice showed a partial preservation of neurotrophic signalling. These data suggested that MPTP disrupts hippocampal neurotrophic pathways in a gender-dependent manner.

**FIGURE 5:**
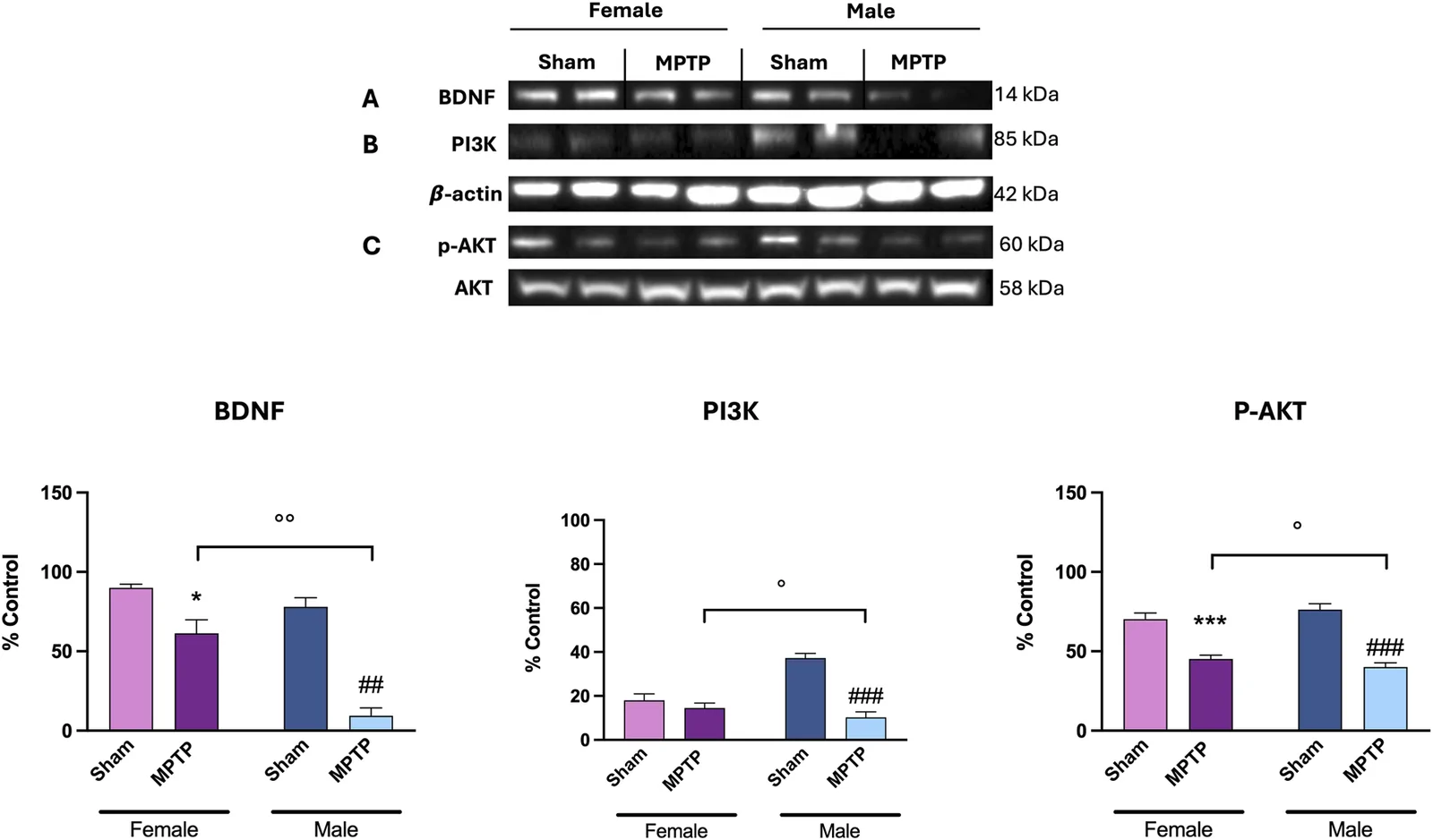
Gender difference on neurotrophic signaling pathway. Western blot for BDNF **(A)**, PI3K **(B)** and p-AKT **(C)**. Data are expressed as mean ± SD. One-way ANOVA test. *p < 0.05 and ***p < 0.001 vs. Female Sham; ##p < 0.01 and ###p < 0.001 vs. Male Sham; °p < 0.05 and °p < 0.01 vs. MPTP.

## 4 Discussion

The present study clearly demonstrated how gender differences influence susceptibility to depressive symptoms in a mouse model of MPTP-induced PD. The results obtained support the hypothesis that the neurobiological mechanisms underlying psychological symptoms in PD are not a uniform phenomenon but exhibit clear sex-specific patterns. Specifically, we observed a greater reduction in TH and DAT and a more marked accumulation of α-syn in males than in females. This observation is consistent with previous studies showing that male subjects are generally more susceptible than females to neurodegeneration and the development of non-motor symptoms in PD (Patel and Kompoliti, 2023; Abraham et al., 2023). A possible protective factor in females is represented by estrogens, known to modulate neuroinflammation, mitochondrial function, and synaptic plasticity (Belvisi et al., 2022). Although our model did not directly assess hormone levels, the observed differences suggest a potential role for hormonal protection, consistent with the hypothesis that women have a more benign course and a tremor-predominant phenotype in the disease. Interestingly, although depressive disorders are generally more prevalent in women, our findings differ from clinical observations showing that women with PD may experience a greater burden of mood-related non-motor symptoms, including depression and anxiety (Cattaneo et al., 2025). However, sex differences observed in patients cannot be directly extrapolated to neurotoxin-based PD models. Indeed, male mice have been reported to show greater susceptibility to MPTP-induced dopaminergic neurotoxicity, possibly also due to the neuroprotective effects of estrogens (Freyaldenhoven et al., 1996; Adamson et al., 2022). Thus, the more pronounced depressive- and anxiety-like phenotype observed in males in our study may partly reflect a greater vulnerability to MPTP-induced nigrostriatal damage. This may be particularly relevant considering that non-motor symptoms in PD arise from the involvement of multiple neural systems beyond the nigrostriatal dopaminergic pathway. Therefore, the increased susceptibility of males to MPTP-induced neurodegeneration may contribute to broader neurobiological alterations underlying the depressive-like phenotype, beyond dopaminergic dysfunction alone. Importantly, our findings extend these observations by showing that this sex-dependent vulnerability is associated with behavioral, neurotransmitter, synaptic, and neurotrophic alterations. Depression represents one of the most common non-motor manifestations of PD and has a profound impact on patients’ quality of life, often complicating therapeutic management. Several studies, conducted with different methodological approaches, have reported a prevalence of depressive symptoms in PD patients, highlighting the strong association between this neurodegenerative disease and depressive disorder (Prange et al., 2022; Bae and Kang, 2022). However, it remains unclear whether such depressive symptoms affect men and women differently. In our model, behavioral tests confirmed that MPTP exposure induced depressive- and anxiety-like behaviors in comparison with healthy mouse controls. Importantly, when responses were analyzed according to gender, both at the behavioral and molecular levels, we observed clear sex-specific differences. Notably, male mice were more likely to develop depressive-like symptoms than females, as demonstrated by behavioral tests (Forced Swim Test, Light/Dark Test, Sucrose Preference Test, and EPM Test). These findings reflect the more pronounced neurodegenerative signs in males. Our results further highlight the involvement of neurotransmitter systems in the pathophysiology of PD-associated depression. The significant reduction in 5-HT2C receptor expression observed in MPTP-treated males suggested a gender-specific vulnerability of the serotonin system. Previous studies have reported that alterations in serotonin signaling, particularly at 5-HT2C receptors, are strongly correlated with emotional dysregulation and the onset of depressive symptoms (Popova et al., 2022). The more pronounced decrease in males may explain their greater susceptibility to depressive-like behaviors, consistent with our behavioral findings. Interestingly, while serum ACH levels remained unchanged, hippocampal ACH levels significantly increased in MPTP-treated mice, especially in males. Hypercholinergic activity has previously been associated with mood disorders and may act synergistically with dopaminergic deficits, exacerbating depressive symptoms (Lester et al., 2010). This finding suggests that an imbalance between dopaminergic and cholinergic signaling may play a key role in mediating sex-specific vulnerability to depression in PD. Overall, these results indicate that the interaction between serotonin dysfunction and cholinergic hyperactivity may represent a critical neurochemical substrate underlying the more pronounced depressive phenotype observed in males. This dual alteration may compromise hippocampal and limbic circuits involved in mood regulation, thus linking neurotransmitter dysregulation to the synaptic and neurotrophic deficits observed in our study. In addition to serotonergic and cholinergic alterations, glutamatergic neurotransmission may represent another relevant component of the neurobiological mechanisms underlying mood-related symptoms in PD. Glutamate plays a key role in synaptic plasticity and in the function of cortico-striatal and limbic circuits, and alterations in glutamatergic signalling have been implicated in both motor and non-motor manifestations of PD (Pagonabarraga et al., 2021). Moreover, modulation of glutamate release has been proposed as a relevant mechanism in PD, as shown by safinamide’s ability to inhibit glutamate release in an experimental model of the disease (Pisano et al., 2020). Thus, glutamatergic dysfunction may contribute to disruption of neuronal circuits involved in emotional regulation, representing an additional link between dopaminergic dysfunction and alterations in synaptic plasticity and neurotrophic signalling associated with depressive symptoms in PD. Synaptic loss is one of the distinctive neuropathological features of PD and substantially contributes to both cognitive decline and mood disturbances associated with the disease. PSD-95, a scaffolding protein located at the postsynaptic density, plays a crucial role in dendritic spine maturation, stabilization, and synaptic plasticity (Kaizuka and Takumi, 2024). SYP, a presynaptic vesicle glycoprotein, regulates vesicle trafficking, synapse formation, and neurotransmitter release (Cousin, 2021). Both PSD-95 and SYP are therefore considered reliable markers of synaptic integrity and connectivity (Shi et al., 2024). Alterations in their expression have been consistently reported in animal models of depression, where reduced PSD-95 levels and impaired SYP responsiveness are associated with deficits in synaptic plasticity and diminished sensitivity to antidepressant treatment. In line with the hypothesis that dendritic and synaptic abnormalities are involved in the pathogenesis of both PD and depression (Jellinger, 2022; Ren and Guo, 2021), our data demonstrate that MPTP-induced nigrostriatal degeneration leads to significant hippocampal synaptic loss compared to healthy controls. This impairment was particularly evident in males, in which PSD-95 and SYP expression was markedly reduced compared to females, suggesting more pronounced synaptic damage and greater neuronal vulnerability. Given the central role of the hippocampus in mood regulation (Jones et al., 2022), the disruption of synaptic integrity in this region may represent a key mechanism underlying the higher susceptibility of males to depressive-like symptoms observed in our model. Finally, to further corroborate these findings, we examined the BDNF/PI3K/AKT signaling pathway in hippocampal lysates, a crucial molecular cascade that regulates neuronal survival, synaptic plasticity, and mood (Li et al., 2023; Bedel and Usta, 2025; Li et al., 2022). Our results showed a significant downregulation of this pathway in MPTP-treated mice, with a more pronounced impairment in males compared to females. The greater dysregulation of the BDNF/PI3K/AKT pathway in males, therefore, provides a mechanistic explanation for their greater vulnerability to depressive-like symptoms, enhancing the concept that sex-specific differences in neurotrophic support are crucial in shaping the non-motor manifestations of PD. Taken together, our results suggested that these molecular alterations are interconnected rather than independent events. Indeed, alongside MPTP-induced nigrostriatal degeneration, characterized by reduced TH and DAT expression and increased α-synuclein accumulation, we observed downregulation of the BDNF/PI3K/AKT signaling pathway. This further compromised neuronal survival and synaptic plasticity, contributing to the reduced expression of PSD-95 and synaptophysin. Therefore, the greater neurodegenerative burden observed in male mice may be linked to altered neurotrophic signaling and synaptic integrity, ultimately contributing to their more pronounced depressive- and anxiety-like phenotype.

## 5 Conclusion

Our study demonstrated that gender differences significantly influence susceptibility to depressive and anxiety symptoms in a mouse model of MPTP-induced PD, with males showing greater vulnerability than females both at the neurodegenerative level and at the behavioral and molecular level, with more pronounced depressive phenotypes, alterations in the serotonergic and cholinergic systems, hippocampal synaptic loss, and dysfunction of the BDNF/PI3K/AKT pathway. These findings suggested that the neurobiological mechanisms underlying the non-motor symptoms of PD are not uniform, but follow distinct gender-related patterns, underscoring the importance of considering sex as a biological variable in understanding and treating PD-associated depression. Although the study has some limitations, including the lack of direct measurements of hormone levels and the fact that the MPTP model does not fully reproduce the complexity of the disease in humans, the results suggested the possibility of developing targeted and sex-specific therapeutic strategies. These approaches could allow for more personalized management of the non-motor symptoms of PD, improving patients’ quality of life and enhancing the efficacy of traditional antidepressant therapies.

## Data Availability

The original contributions presented in the study are included in the article/supplementary material, further inquiries can be directed to the corresponding author.
